# Supplementary material for: AI modeling for outbreak prediction: A graph-neural-network approach for identifying vancomycin-resistant enterococcus carriers
Source: PLOS Digit Health. 2025 Apr 10;4(4):e0000821. doi: 10.1371/journal.pdig.0000821 (PMC11984732; doi:10.1371/journal.pdig.0000821)
Supplement: S1 Table — S1 includes macro F1 scores; sensitivity; specificity; precision; recall; accuracy; negative predictive value; positive predictive value; standard deviations from cross-validation or time-curve shifts; p-values for all evaluated model setups and feature combinations. (PDF) [file pdig.0000821.s001.pdf]

S1 Table

Values from the cross validation (dynamic model) or from the temporal split of the data (dynamic model) for the parameters accuracy, precision, recall, F1, sensitivity, specificity, positive predictive value, and negative predictive value and statistical comparison of various metrics with the Mann-Whitney-U-test of the two models, the lengths of the training times and the feature combinations. Significant values are highlighted in red.

Static model:

no features								
	**Accuracy**	**Precision**	**Recall**	**F1**	**Sensitivity**	**Specificity**	**Positive predictive value**	**Negative predictive value**
cross validation data split 1	0.547170	0.578074	0.606914	0.526786	0.720000	0.493827	0.305085	0.851064
cross validation data split 2	0.466667	0.596667	0.608750	0.465455	0.880000	0.337500	0.293333	0.900000
cross validation data split 3	0.552381	0.628205	0.665000	0.544279	0.880000	0.450000	0.333333	0.923077
cross validation data split 4	0.580952	0.604167	0.642500	0.559832	0.760000	0.525000	0.333333	0.875000
cross validation data split 5	0.390476	0.640449	0.600000	0.385965	1.000000	0.200000	0.280899	1.000000
mean value	0.507529	0.609512	0.624633	0.496463	0.848000	0.401265	0.309197	0.909828
standard deviation	0.069778	0.022309	0.024987	0.063864	0.099277	0.119018	0.021140	0.051126

age and sex								
	**Accuracy**	**Precision**	**Recall**	**F1**	**Sensitivity**	**Specificity**	**Positive predictive value**	**Negative predictive value**
cross validation data split 1	0.471698	0.583230	0.599012	0.468672	0.840000	0.358025	0.287671	0.878788
cross validation data split 2	0.457143	0.610399	0.616250	0.456946	0.920000	0.312500	0.294872	0.925926
cross validation data split 3	0.485714	0.603810	0.621250	0.483418	0.880000	0.362500	0.301370	0.906250
cross validation data split 4	0.457143	0.550000	0.561250	0.452174	0.760000	0.362500	0.271429	0.828571
cross validation data split 5	0.400000	0.589286	0.578750	0.399128	0.920000	0.237500	0.273810	0.904762
mean value	0.454340	0.587345	0.595302	0.452068	0.864000	0.326605	0.285830	0.888859
standard deviation	0.029169	0.021061	0.022625	0.028583	0.059867	0.048379	0.011649	0.033661

level of care, information about falls and open wounds								
	**Accuracy**	**Precision**	**Recall**	**F1**	**Sensitivity**	**Specificity**	**Positive predictive value**	**Negative predictive value**
cross validation data split 1	0.688679	0.615873	0.644198	0.620237	0.560000	0.728395	0.388889	0.842857
cross validation data split 2	0.638095	0.584794	0.611250	0.580177	0.560000	0.662500	0.341463	0.828125
cross validation data split 3	0.590476	0.587990	0.621250	0.559125	0.680000	0.562500	0.326923	0.849057
cross validation data split 4	0.619048	0.564787	0.585000	0.558081	0.520000	0.650000	0.317073	0.812500
cross validation data split 5	0.580952	0.544444	0.560000	0.528571	0.520000	0.600000	0.288889	0.800000
mean value	0.623450	0.579578	0.604340	0.569238	0.568000	0.640679	0.332647	0.826508
standard deviation	0.038424	0.023952	0.029197	0.030335	0.058788	0.056611	0.032957	0.018315

ICD codes (diagnoses)								
	**Accuracy**	**Precision**	**Recall**	**F1**	**Sensitivity**	**Specificity**	**Positive predictive value**	**Negative predictive value**
cross validation data split 1	0.726415	0.651144	0.682716	0.659466	0.600000	0.765432	0.441176	0.861111
cross validation data split 2	0.742857	0.698413	0.762500	0.704102	0.800000	0.725000	0.476190	0.920635
cross validation data split 3	0.695238	0.636946	0.676250	0.640411	0.640000	0.712500	0.410256	0.863636
cross validation data split 4	0.714286	0.664825	0.716250	0.668561	0.720000	0.712500	0.439024	0.890625
cross validation data split 5	0.733333	0.670906	0.715000	0.679599	0.680000	0.750000	0.459459	0.882353
mean value	0.722426	0.664447	0.710543	0.670428	0.688000	0.733086	0.445221	0.883672
standard deviation	0.016479	0.020634	0.030667	0.021179	0.068819	0.021191	0.022087	0.021572

Braden								
	**Accuracy**	**Precision**	**Recall**	**F1**	**Sensitivity**	**Specificity**	**Positive predictive value**	**Negative predictive value**
cross validation data split 1	0.556604	0.582256	0.613086	0.534697	0.720000	0.506173	0.310345	0.854167
cross validation data split 2	0.438095	0.528571	0.535000	0.432952	0.720000	0.350000	0.257143	0.800000
cross validation data split 3	0.514286	0.552778	0.571250	0.496000	0.680000	0.462500	0.283333	0.822222
cross validation data split 4	0.533333	0.572222	0.597500	0.515765	0.720000	0.475000	0.300000	0.844444
cross validation data split 5	0.457143	0.630000	0.630000	0.457143	0.960000	0.300000	0.300000	0.960000
mean value	0.499892	0.573165	0.589367	0.487311	0.760000	0.418735	0.290164	0.856167
standard deviation	0.045140	0.033817	0.033358	0.037405	0.101193	0.079434	0.018643	0.055190

bedsore								
	**Accuracy**	**Precision**	**Recall**	**F1**	**Sensitivity**	**Specificity**	**Positive predictive value**	**Negative predictive value**
cross validation data split 1	0.518868	0.601780	0.629877	0.511521	0.840000	0.419753	0.308824	0.894737
cross validation data split 2	0.600000	0.634488	0.682500	0.583333	0.840000	0.525000	0.355932	0.913043
cross validation data split 3	0.514286	0.614286	0.640000	0.509840	0.880000	0.400000	0.314286	0.914286
cross validation data split 4	0.571429	0.611111	0.650000	0.555294	0.800000	0.500000	0.333333	0.888889
cross validation data split 5	0.380952	0.606952	0.580000	0.377338	0.960000	0.200000	0.272727	0.941176
mean value	0.517107	0.613723	0.636475	0.507465	0.864000	0.408951	0.317020	0.910426
standard deviation	0.075328	0.011196	0.033307	0.070709	0.054259	0.114555	0.027629	0.018316

bedsore_admission								
	Accuracy	Precision	Recall	F1	Sensitivity	Specificity	Positive predictive value	Negative predictive value
cross validation data split 1	0.481132	0.587010	0.605185	0.477364	0.840000	0.370370	0.291667	0.882353
cross validation data split 2	0.704762	0.643362	0.682500	0.648526	0.640000	0.725000	0.421053	0.865672
cross validation data split 3	0.523810	0.617754	0.646250	0.518525	0.880000	0.412500	0.318841	0.916667
cross validation data split 4	0.495238	0.567016	0.586250	0.486102	0.760000	0.412500	0.287879	0.846154
cross validation data split 5	0.371429	0.576628	0.560000	0.368622	0.920000	0.200000	0.264368	0.888889
mean value	0.515274	0.598354	0.616037	0.499828	0.808000	0.424074	0.316761	0.879947
standard deviation	0.107923	0.028231	0.043532	0.089854	0.099277	0.169657	0.054936	0.023579

Barthel								
	Accuracy	Precision	Recall	F1	Sensitivity	Specificity	Positive predictive value	Negative predictive value
cross validation data split 1	0.556604	0.561610	0.585432	0.525840	0.640000	0.530864	0.296296	0.826923
cross validation data split 2	0.714286	0.642857	0.675000	0.650000	0.600000	0.750000	0.428571	0.857143
cross validation data split 3	0.580952	0.573529	0.601250	0.546347	0.640000	0.562500	0.313725	0.833333
cross validation data split 4	0.571429	0.559091	0.581250	0.533333	0.600000	0.562500	0.300000	0.818182
cross validation data split 5	0.533333	0.572222	0.597500	0.515765	0.720000	0.475000	0.300000	0.844444
mean value	0.591321	0.581862	0.608086	0.554257	0.640000	0.576173	0.327719	0.836005
standard deviation	0.063547	0.031021	0.034263	0.048900	0.043818	0.092605	0.050776	0.013604

Barthel_extended

	Accuracy	Precision	Recall	F1	Sensitivity	Specificity	Positive predictive value	Negative predictive value
cross validation data split 1	0.566038	0.632645	0.674568	0.555920	0.880000	0.469136	0.338462	0.926829
cross validation data split 2	0.704762	0.588735	0.586250	0.587400	0.360000	0.812500	0.375000	0.802469
cross validation data split 3	0.514286	0.587413	0.612500	0.505495	0.800000	0.425000	0.303030	0.871795
cross validation data split 4	0.561905	0.607116	0.643750	0.547074	0.800000	0.487500	0.327869	0.886364
cross validation data split 5	0.400000	0.613219	0.592500	0.398034	0.960000	0.225000	0.279070	0.947368
mean value	0.549398	0.605826	0.621914	0.518785	0.760000	0.483827	0.324686	0.886965
standard deviation	0.098106	0.016774	0.033091	0.065795	0.208614	0.189054	0.032500	0.050215

OPS codes (operations, procedures)

	Accuracy	Precision	Recall	F1	Sensitivity	Specificity	Positive predictive value	Negative predictive value
cross validation data split 1	0.716981	0.643863	0.676543	0.651316	0.600000	0.753086	0.428571	0.859155
cross validation data split 2	0.714286	0.672543	0.730000	0.673778	0.760000	0.700000	0.441860	0.903226
cross validation data split 3	0.695238	0.644817	0.690000	0.646465	0.680000	0.700000	0.414634	0.875000
cross validation data split 4	0.723810	0.678571	0.736250	0.682183	0.760000	0.712500	0.452381	0.904762
cross validation data split 5	0.733333	0.664286	0.701250	0.673333	0.640000	0.762500	0.457143	0.871429
mean value	0.716730	0.660816	0.706809	0.665415	0.688000	0.725617	0.438918	0.882714
standard deviation	0.012599	0.014200	0.022952	0.013941	0.064000	0.026831	0.015613	0.018159

clinical chemistry data from blood								
	Accuracy	Precision	Recall	F1	Sensitivity	Specificity	Positive predictive value	Negative predictive value
cross validation data split 1	0.603774	0.593939	0.630123	0.569272	0.680000	0.580247	0.333333	0.854545
cross validation data split 2	0.619048	0.642544	0.695000	0.599848	0.840000	0.550000	0.368421	0.916667
cross validation data split 3	0.476190	0.571429	0.587500	0.471396	0.800000	0.375000	0.285714	0.857143
cross validation data split 4	0.495238	0.579491	0.600000	0.488558	0.800000	0.400000	0.294118	0.864865
cross validation data split 5	0.533333	0.607809	0.638750	0.524887	0.840000	0.437500	0.318182	0.897436
mean value	0.545517	0.599042	0.630275	0.530792	0.792000	0.468549	0.319954	0.878131
standard deviation	0.057067	0.025060	0.037436	0.048158	0.058788	0.081884	0.029567	0.024619

ICD and OPS codes								
	Accuracy	Precision	Recall	F1	Sensitivity	Specificity	Positive predictive value	Negative predictive value
cross validation data split 1	0.726415	0.657937	0.696543	0.666269	0.640000	0.753086	0.444444	0.871429
cross validation data split 2	0.800000	0.730000	0.758750	0.741349	0.680000	0.837500	0.566667	0.893333
cross validation data split 3	0.723810	0.643408	0.667500	0.650843	0.560000	0.775000	0.437500	0.849315
cross validation data split 4	0.761905	0.693662	0.733750	0.705354	0.680000	0.787500	0.500000	0.887324
cross validation data split 5	0.771429	0.692831	0.712500	0.700855	0.600000	0.825000	0.517241	0.868421
mean value	0.756712	0.683567	0.713809	0.692934	0.632000	0.795617	0.493170	0.873964
standard deviation	0.028698	0.030376	0.031182	0.031761	0.046648	0.031360	0.047961	0.015481

all features combined								
	**Accuracy**	**Precision**	**Recall**	**F1**	**Sensitivity**	**Specificity**	**Positive predictive value**	**Negative predictive value**
cross validation data split 1	0.726415	0.657937	0.696543	0.666269	0.640000	0.753086	0.444444	0.871429
cross validation data split 2	0.780952	0.706667	0.732500	0.716716	0.640000	0.825000	0.533333	0.880000
cross validation data split 3	0.723810	0.643408	0.667500	0.650843	0.560000	0.775000	0.437500	0.849315
cross validation data split 4	0.752381	0.685714	0.727500	0.696667	0.680000	0.775000	0.485714	0.885714
cross validation data split 5	0.761905	0.683333	0.706250	0.692082	0.600000	0.812500	0.500000	0.866667
mean value	0.749093	0.675412	0.706059	0.684515	0.624000	0.788117	0.480198	0.870625
standard deviation	0.021650	0.022250	0.023393	0.023275	0.040792	0.026556	0.035630	0.012538

Dynamic model:

no features								
	**Accuracy**	**Precision**	**Recall**	**F1**	**Sensitivity**	**Specificity**	**Positive predictive value**	**Negative predictive value**
temporal data split 1	0.696296	0.713356	0.677854	0.674856	0.486339	0.869369	0.754237	0.672474
temporal data split 2	0.720824	0.705562	0.690543	0.695070	0.559524	0.821561	0.661972	0.749153
temporal data split 3	0.724051	0.757072	0.681831	0.681755	0.432927	0.930736	0.816092	0.698052
temporal data split 4	0.671470	0.675170	0.644653	0.642359	0.446667	0.842640	0.683673	0.666667
temporal data split 5	0.708215	0.688015	0.651734	0.657356	0.441860	0.861607	0.647727	0.728302
mean value	0.704171	0.707835	0.669323	0.670279	0.473463	0.865183	0.712740	0.702929
standard deviation	0.019080	0.027999	0.017875	0.018514	0.046759	0.036711	0.063329	0.031770

age and sex								
	**Accuracy**	**Precision**	**Recall**	**F1**	**Sensitivity**	**Specificity**	**Positive predictive value**	**Negative predictive value**
temporal data split 1	0.711111	0.719998	0.696647	0.696796	0.546448	0.846847	0.746269	0.693727
temporal data split 2	0.675057	0.654399	0.648898	0.650880	0.535714	0.762082	0.584416	0.724382
temporal data split 3	0.701266	0.691973	0.686226	0.688152	0.597561	0.774892	0.653333	0.730612
temporal data split 4	0.706052	0.701755	0.692606	0.694669	0.593333	0.791878	0.684615	0.718894
temporal data split 5	0.725212	0.705494	0.679921	0.686532	0.511628	0.848214	0.660000	0.750988
mean value	0.703740	0.694724	0.680860	0.683406	0.556937	0.804783	0.665727	0.723721
standard deviation	0.016429	0.022081	0.016961	0.016712	0.033432	0.036164	0.052233	0.018517

level of care, information about falls and open wounds								
	**Accuracy**	**Precision**	**Recall**	**F1**	**Sensitivity**	**Specificity**	**Positive predictive value**	**Negative predictive value**
temporal data split 1	0.725926	0.735513	0.712081	0.712984	0.568306	0.855856	0.764706	0.706320
temporal data split 2	0.663616	0.643116	0.640722	0.641733	0.541667	0.739777	0.565217	0.721014
temporal data split 3	0.751899	0.748943	0.733054	0.737339	0.621951	0.844156	0.739130	0.758755
temporal data split 4	0.714697	0.716473	0.695448	0.697823	0.553333	0.837563	0.721739	0.711207
temporal data split 5	0.722380	0.705634	0.715497	0.708510	0.689922	0.741071	0.605442	0.805825
mean value	0.715703	0.709936	0.699360	0.699678	0.595036	0.803685	0.679247	0.740624
standard deviation	0.028891	0.036611	0.031658	0.031731	0.054827	0.051985	0.078924	0.037434

ICD codes (diagnoses)								
	**Accuracy**	**Precision**	**Recall**	**F1**	**Sensitivity**	**Specificity**	**Positive predictive value**	**Negative predictive value**
temporal data split 1	0.839506	0.847933	0.831044	0.834850	0.743169	0.918919	0.883117	0.812749
temporal data split 2	0.867277	0.862308	0.855317	0.858472	0.803571	0.907063	0.843750	0.880866
temporal data split 3	0.863291	0.868563	0.849514	0.855980	0.768293	0.930736	0.887324	0.849802
temporal data split 4	0.899135	0.900503	0.893672	0.896438	0.853333	0.934010	0.907801	0.893204
temporal data split 5	0.849858	0.899044	0.796217	0.818874	0.596899	0.995536	0.987179	0.810909
mean value	0.863814	0.875670	0.845153	0.852923	0.753053	0.937253	0.901834	0.849506
standard deviation	0.020226	0.020791	0.031844	0.026152	0.086397	0.030651	0.047443	0.033864

Braden								
	Accuracy	Precision	Recall	F1	Sensitivity	Specificity	Positive predictive value	Negative predictive value
temporal data split 1	0.698765	0.702853	0.685866	0.686214	0.551913	0.819820	0.716312	0.689394
temporal data split 2	0.736842	0.729397	0.697966	0.704787	0.529762	0.866171	0.712000	0.746795
temporal data split 3	0.764557	0.774160	0.737686	0.743973	0.579268	0.896104	0.798319	0.750000
temporal data split 4	0.734870	0.735519	0.718782	0.721878	0.600000	0.837563	0.737705	0.733333
temporal data split 5	0.728045	0.721219	0.665715	0.672845	0.434109	0.897321	0.708861	0.733577
mean value	0.732616	0.732630	0.701203	0.705939	0.539010	0.863396	0.734639	0.730620
standard deviation	0.021023	0.023496	0.025080	0.025244	0.057614	0.030967	0.033399	0.021693

bedsore								
	Accuracy	Precision	Recall	F1	Sensitivity	Specificity	Positive predictive value	Negative predictive value
temporal data split 1	0.698765	0.708797	0.682986	0.681997	0.519126	0.846847	0.736434	0.681159
temporal data split 2	0.704805	0.687229	0.674179	0.678040	0.541667	0.806691	0.636364	0.738095
temporal data split 3	0.751899	0.759867	0.724211	0.729784	0.560976	0.887446	0.779661	0.740072
temporal data split 4	0.662824	0.663978	0.636244	0.633600	0.440000	0.832487	0.666667	0.661290
temporal data split 5	0.696884	0.674020	0.675682	0.674794	0.596899	0.754464	0.583333	0.764706
mean value	0.703036	0.698778	0.678660	0.679643	0.531733	0.825587	0.680492	0.717065
standard deviation	0.028509	0.034028	0.028006	0.030548	0.052487	0.044140	0.070060	0.039093

bedsore_admission

	Accuracy	Precision	Recall	F1	Sensitivity	Specificity	Positive predictive value	Negative predictive value
temporal data split 1	0.693827	0.702011	0.678482	0.677548	0.519126	0.837838	0.725191	0.678832
temporal data split 2	0.723112	0.709554	0.689049	0.694376	0.541667	0.836431	0.674074	0.745033
temporal data split 3	0.736709	0.732881	0.716529	0.720544	0.597561	0.835498	0.720588	0.745174
temporal data split 4	0.668588	0.672206	0.641320	0.638571	0.440000	0.842640	0.680412	0.664000
temporal data split 5	0.702550	0.679562	0.680146	0.679848	0.596899	0.763393	0.592308	0.766816
mean value	0.704957	0.699243	0.681105	0.682177	0.539051	0.823160	0.678515	0.719971
standard deviation	0.023623	0.021743	0.024119	0.026643	0.058283	0.029985	0.047757	0.040701

Barthel

	Accuracy	Precision	Recall	F1	Sensitivity	Specificity	Positive predictive value	Negative predictive value
temporal data split 1	0.688889	0.706672	0.669657	0.665544	0.469945	0.869369	0.747826	0.665517
temporal data split 2	0.707094	0.690357	0.673803	0.678174	0.529762	0.817844	0.644928	0.735786
temporal data split 3	0.774684	0.776474	0.754303	0.759905	0.634146	0.874459	0.781955	0.770992
temporal data split 4	0.708934	0.724185	0.681624	0.681405	0.480000	0.883249	0.757895	0.690476
temporal data split 5	0.583569	0.541070	0.538725	0.538784	0.372093	0.705357	0.421053	0.661088
mean value	0.692634	0.687752	0.663622	0.664762	0.497189	0.830056	0.670731	0.704772
standard deviation	0.061832	0.078841	0.069694	0.071246	0.085446	0.066401	0.133354	0.042428

Barthel_extended								
	**Accuracy**	**Precision**	**Recall**	**F1**	**Sensitivity**	**Specificity**	**Positive predictive value**	**Negative predictive value**
temporal data split 1	0.691358	0.695081	0.678149	0.678165	0.540984	0.815315	0.707143	0.683019
temporal data split 2	0.723112	0.709554	0.689049	0.694376	0.541667	0.836431	0.674074	0.745033
temporal data split 3	0.723112	0.709554	0.689049	0.694376	0.541667	0.836431	0.674074	0.745033
temporal data split 4	0.688761	0.692525	0.664653	0.664674	0.486667	0.842640	0.701923	0.683128
temporal data split 5	0.682720	0.655372	0.649727	0.651923	0.527132	0.772321	0.571429	0.739316
mean value	0.701813	0.692417	0.674125	0.676703	0.527623	0.820628	0.665729	0.719106
standard deviation	0.017616	0.019833	0.015144	0.016646	0.021216	0.025868	0.049106	0.029494

OPS codes (operations, procedures)								
	**Accuracy**	**Precision**	**Recall**	**F1**	**Sensitivity**	**Specificity**	**Positive predictive value**	**Negative predictive value**
temporal data split 1	0.846914	0.847269	0.843081	0.844639	0.803279	0.882883	0.849711	0.844828
temporal data split 2	0.883295	0.874870	0.881738	0.877883	0.875000	0.888476	0.830508	0.919231
temporal data split 3	0.911392	0.910457	0.906557	0.908347	0.878049	0.935065	0.905660	0.915254
temporal data split 4	0.922190	0.928249	0.914772	0.919624	0.860000	0.969543	0.955556	0.900943
temporal data split 5	0.875354	0.905272	0.834389	0.854733	0.682171	0.986607	0.967033	0.843511
mean value	0.887829	0.893224	0.876107	0.881045	0.819700	0.932515	0.901694	0.884753
standard deviation	0.026781	0.028697	0.032511	0.029194	0.073852	0.041729	0.054691	0.033693

clinical chemistry data from blood								
	**Accuracy**	**Precision**	**Recall**	**F1**	**Sensitivity**	**Specificity**	**Positive predictive value**	**Negative predictive value**
temporal data split 1	0.753086	0.765266	0.739736	0.741709	0.601093	0.878378	0.802920	0.727612
temporal data split 2	0.679634	0.661555	0.661555	0.661555	0.583333	0.739777	0.583333	0.739777
temporal data split 3	0.800000	0.794161	0.793633	0.793892	0.756098	0.831169	0.760736	0.827586
temporal data split 4	0.780980	0.788876	0.764162	0.769007	0.640000	0.888325	0.813559	0.764192
temporal data split 5	0.787535	0.772917	0.761905	0.766479	0.666667	0.857143	0.728814	0.817021
mean value	0.760247	0.756555	0.744198	0.746528	0.649438	0.838958	0.737872	0.775238
standard deviation	0.043138	0.048636	0.044734	0.045586	0.060777	0.053328	0.083002	0.040333

days since admission								
	**Accuracy**	**Precision**	**Recall**	**F1**	**Sensitivity**	**Specificity**	**Positive predictive value**	**Negative predictive value**
temporal data split 1	0.659259	0.659883	0.645510	0.644321	0.502732	0.788288	0.661871	0.657895
temporal data split 2	0.778032	0.765829	0.771652	0.768200	0.744048	0.799257	0.698324	0.833333
temporal data split 3	0.736709	0.728909	0.728909	0.728909	0.682927	0.774892	0.682927	0.774892
temporal data split 4	0.697406	0.692714	0.683401	0.685322	0.580000	0.786802	0.674419	0.711009
temporal data split 5	0.742210	0.722180	0.722903	0.722535	0.651163	0.794643	0.646154	0.798206
mean value	0.722723	0.713903	0.710475	0.709857	0.632174	0.788776	0.672739	0.755067
standard deviation	0.040750	0.035657	0.042873	0.042011	0.083582	0.008260	0.017811	0.062870

ICD and OPS codes								
	**Accuracy**	**Precision**	**Recall**	**F1**	**Sensitivity**	**Specificity**	**Positive predictive value**	**Negative predictive value**
temporal data split 1	0.876543	0.892857	0.866748	0.872159	0.765027	0.968468	0.952381	0.833333
temporal data split 2	0.903890	0.899949	0.896232	0.898002	0.863095	0.929368	0.884146	0.915751
temporal data split 3	0.881013	0.882233	0.871740	0.875923	0.817073	0.926407	0.887417	0.877049
temporal data split 4	0.922190	0.928249	0.914772	0.919624	0.860000	0.969543	0.955556	0.900943
temporal data split 5	0.878187	0.915073	0.834977	0.856954	0.674419	0.995536	0.988636	0.841509
mean value	0.892365	0.903672	0.876894	0.884532	0.795923	0.957864	0.933627	0.873717
standard deviation	0.017903	0.016276	0.027185	0.021912	0.070403	0.026342	0.041091	0.032210

all features combined								
	**Accuracy**	**Precision**	**Recall**	**F1**	**Sensitivity**	**Specificity**	**Positive predictive value**	**Negative predictive value**
temporal data split 1	0.849383	0.858411	0.841013	0.845014	0.754098	0.927928	0.896104	0.820717
temporal data split 2	0.897025	0.888835	0.905182	0.893813	0.940476	0.869888	0.818653	0.959016
temporal data split 3	0.886076	0.887594	0.876953	0.881203	0.823171	0.930736	0.894040	0.881148
temporal data split 4	0.927954	0.937624	0.919052	0.925250	0.853333	0.984772	0.977099	0.898148
temporal data split 5	0.875354	0.913426	0.831101	0.853250	0.666667	0.995536	0.988506	0.838346
mean value	0.887158	0.897178	0.874660	0.879706	0.807549	0.941772	0.914880	0.879475
standard deviation	0.025804	0.026699	0.034459	0.028910	0.092428	0.045211	0.062191	0.048626

Training periode:

30 days<br>all features combined								
	**Accuracy**	**Precision**	**Recall**	**F1**	**Sensitivity**	**Specificity**	**Positive predictive value**	**Negative predictive value**
temporal data split 1	0.849383	0.858411	0.841013	0.845014	0.754098	0.927928	0.896104	0.820717
temporal data split 2	0.897025	0.888835	0.905182	0.893813	0.940476	0.869888	0.818653	0.959016
temporal data split 3	0.886076	0.887594	0.876953	0.881203	0.823171	0.930736	0.894040	0.881148
temporal data split 4	0.927954	0.937624	0.919052	0.925250	0.853333	0.984772	0.977099	0.898148
temporal data split 5	0.875354	0.913426	0.831101	0.853250	0.666667	0.995536	0.988506	0.838346
mean value	0.887158	0.897178	0.874660	0.879706	0.807549	0.941772	0.914880	0.879475
standard deviation	0.025804	0.026699	0.034459	0.028910	0.092428	0.045211	0.062191	0.048626

60 days<br>all features combined								
	**Accuracy**	**Precision**	**Recall**	**F1**	**Sensitivity**	**Specificity**	**Positive predictive value**	**Negative predictive value**
temporal data split 1	0.834568	0.849856	0.823660	0.828195	0.710383	0.936937	0.902778	0.796935
temporal data split 2	0.876430	0.867611	0.881749	0.872255	0.904762	0.858736	0.800000	0.935223
temporal data split 3	0.903797	0.908182	0.893873	0.899354	0.835366	0.952381	0.925676	0.890688
temporal data split 4	0.930836	0.939799	0.922386	0.928321	0.860000	0.984772	0.977273	0.902326
temporal data split 5	0.903683	0.930392	0.869861	0.889362	0.744186	0.995536	0.989691	0.871094
mean value	0.889863	0.899168	0.878306	0.883497	0.810939	0.945672	0.919083	0.879253
standard deviation	0.032563	0.035027	0.032418	0.033105	0.072628	0.048362	0.067619	0.046127

90 days<br>all features combined								
	**Accuracy**	**Precision**	**Recall**	**F1**	**Sensitivity**	**Specificity**	**Positive predictive value**	**Negative predictive value**
temporal data split 1	0.859259	0.870280	0.850502	0.854925	0.759563	0.941441	0.914474	0.826087
temporal data split 2	0.860412	0.850944	0.862033	0.855056	0.869048	0.855019	0.789189	0.912698
temporal data split 3	0.896203	0.895440	0.890033	0.892427	0.853659	0.926407	0.891720	0.899160
temporal data split 4	0.927954	0.937624	0.919052	0.925250	0.853333	0.984772	0.977099	0.898148
temporal data split 5	0.895184	0.914783	0.863164	0.880430	0.744186	0.982143	0.960000	0.869565
mean value	0.887802	0.893814	0.876957	0.881618	0.815958	0.937956	0.906496	0.881132
standard deviation	0.025699	0.030833	0.024722	0.026230	0.052855	0.047262	0.066167	0.030902

Statistic tests:

Static model vs dynamic model

	p value							
**Feature**	**Accuracy**	**Precision**	**Recall**	**F1**	**Sensitivity**	**Specificity**	**Positive predictive value**	**Negative predictive value**
**no features**	0.008	0.008	0.032	0.008	0.008	0.008	0.008	0.008
**age and sex**	0.008	0.008	0.008	0.008	0.008	0.008	0.008	0.008
**level of care, information about falls and open wounds**	0.016	0.008	0.016	0.008	0.421	0.008	0.008	0.016
**ICD codes (diagnoses)**	0.008	0.008	0.008	0.008	0.310	0.008	0.008	0.222
**Braden**	0.008	0.008	0.008	0.008	0.008	0.008	0.008	0.008
**Bedsore**	0.008	0.008	0.151	0.008	0.008	0.008	0.008	0.008
**Bedsore_admission**	0.056	0.008	0.095	0.016	0.008	0.008	0.008	0.008
**Barthel**	0.095	0.151	0.310	0.032	0.032	0.016	0.016	0.008
**Barthel_extended**	0.056	0.008	0.032	0.008	0.151	0.016	0.008	0.008
**OPS codes (operations, procedures)**	0.008	0.008	0.008	0.008	0.032	0.008	0.008	1.000
**clinical chemistry data from blood**	0.008	0.008	0.016	0.008	0.016	0.008	0.008	0.008
**ICD and OPS codes**	0.008	0.008	0.008	0.008	0.032	0.008	0.008	1.000
**all features combined**	0.008	0.008	0.008	0.008	0.016	0.008	0.008	0.841

Training periode

	p value							
	Accuracy	Precision	Recall	F1	Sensitivity	Specificity	Positive predictive value	Negative predictive value
30 vs 60	0.690	1.000	1.000	0.841	1.000	0.841	0.690	1.000
30 vs 90	1.000	1.000	0.841	0.841	0.690	0.690	0.690	0.690

Used features dynamic model

	p value							
	Accuracy	Precision	Recall	F1	Sensitivity	Specificity	Positive predictive value	Negative predictive value
no features vs age and sex	1.000	0.690	0.310	0.310	0.056	0.095	0.421	0.421
no features vs level of care, information about falls and open wounds	0.421	0.690	0.151	0.151	0.032	0.151	0.690	0.222
no features vs ICD codes (diagnoses)	0.008	0.008	0.008	0.008	0.008	0.032	0.008	0.008
no features vs Braden	0.056	0.222	0.095	0.095	0.222	1.000	0.548	0.222
no features vs bedsore	0.841	0.548	1.000	0.841	0.222	0.310	0.548	0.690
no features vs bedsore_admission	1.000	0.690	0.690	0.690	0.222	0.950	0.690	0.690
no features vs Barthel	0.841	0.841	1.000	1.000	0.690	0.841	0.841	0.841
no features vs Barthel_extended	0.841	0.690	0.841	0.841	0.151	0.056	0.690	0.548
no features vs OPS codes (operations, procedures)	0.008	0.008	0.008	0.008	0.032	0.008	0.008	0.008
no features vs clinical chemistry data from blood	0.421	0.690	0.151	0.151	0.016	0.008	0.421	0.310
no features vs days since admission	0.095	0.151	0.056	0.056	0.008	0.841	0.690	0.056
no features vs all features combined	0.008	0.008	0.008	0.008	0.008	0.032	0.008	0.008
all features vs ICD codes	0.310	0.421	0.310	0.421	0.421	1.000	0.690	0.310
all features vs OPS codes	0.841	0.841	0.841	1.000	0.690	1.000	0.841	0.548
all features vs OPS and ICD codes	0.841	0.690	1.000	0.841	1.000	0.841	1.000	1.000
