# Supplementary material for: AI modeling for outbreak prediction: A graph-neural-network approach for identifying vancomycin-resistant enterococcus carriers
Source: PLOS Digit Health. 2025 Apr 10;4(4):e0000821. doi: 10.1371/journal.pdig.0000821 (PMC11984732; doi:10.1371/journal.pdig.0000821)
Supplement: Attachment — Submitted filename: Response_to_Reviewers.docx [file pdig.0000821.s003.docx]

PLOS Digital Health

Sulaf Assi

Academic Editor

Regensburg, 19.02.2025

**PDIG-D-24-00384**

Revised version of the manuscript **AI Modeling for Outbreak Prediction: A Graph-Neural-Network Approach for Identifying Vancomycin-Resistant Enterococcus Carriers**

Dear Sulaf Assi,

On behalf of my co-authors, I would like to thank you and the reviewers for your valuable comments and suggestions to improve the quality of our manuscript. We have revised the manuscript according to the reviewers' comments. Enclosed, you will find a point-by-point response to each comment, indicating the actions taken or the revisions made.

In addition to this letter, we have uploaded a version of the revised manuscript with tracked changes ('Revised Manuscript with Track Changes') and a version without tracked changes ('Manuscript').

Thank you very much again for your help in improving the quality of our paper. We trust that our manuscript is now suitable for publication in PLOS Digital Health.

Sincerely,

Dr. Bärbel Kieninger

Reviewer #1: The paper presents a new approach to predicting VRE carriers using artificial intelligence. The reviewer believes there are some important drawbacks and weakness that should be considered to improve the quality of the paper.

1. A clearer statement of the study's significance or implications for clinical practice should be provided in Abstract. It lacks a brief mention of the potential impact on patient safety or infection control strategies.

Thank you for your feedback! We have added the following to the abstract:

Line 44ff:

Such systems could help enhance patient safety and proactively reduce nosocomial transmission events through targeted, cost-efficient interventions. Moreover, they could enable a more effective approach to managing antimicrobial resistance.

We have added the following to the discussion:

Line 332ff:

It is important for us to emphasize that our project, made possible only through the digitization of patient records, aims to give this documentation a broader purpose by actively utilizing the resulting dataset to improve diagnostics, therapy, and patient care. In the future, we hope to contribute to evidence-based data utilization for the benefit of patients and to support efforts in combating multidrug-resistant pathogens and antibiotic resistance.

2. More context on the global impact of VRE infections, including statistics on morbidity and mortality, to emphasize urgency can be added to Introduction to outlines the problem of VRE.

We have supplemented and modified the introduction. The text now reads as follows:

Line 82ff:

According to the WHO Bacterial Priority Pathogen List^1^, vancomycin-resistant enterococci (VRE) are a significant threat to patient safety and causing a growing burden of disease. In Europe. A rising number of reported enterococcal bloodstream infections (BSI; +57.3%) – and especially of VRE SBI (+80.0%) – was reported between 2017-2021^2^. Moreover, in 2019, 200,000 deaths worldwide were attributable to or associated with VRE^3^.

VRE are Gram-positive cocci found in the human gut. Infections - like bloodstream or urinary tract infections - commonly occur subsequent to colonization and lead to prolonged hospitalization, increase patient suffering and significantly worsen the prognosis of affected patients^4^. A European systematic review analyzing data over a 10-year period reported a mortality rate of 33.5%^5,6^. Certain patient characteristics such as bone marrow transplant, central venous catheter, bedsores, and concurrent *Clostridioides difficile* infection, increase the susceptibility of patients to VRE^7^. Therefore, the development of effective strategies to slow down the spread of VRE and prevent their nosocomial transmission is crucial to avoiding additional risks, particularly for these critically ill patients.

Our current knowledge is that nosocomial VRE transmission occurs mainly through direct contact or through fomites, with healthcare workers' hands being the primary vector. VRE are highly stable in the environment and can survive for months in dry conditions^8-14^. When surfaces are not properly decontaminated, they act as permanent reservoirs for VRE, affecting not only the initial patient but also subsequent patients in the same room or nearby^15-17^. This knowledge has led to the consideration that reduction of shared surfaces between VRE colonized and non-colonized patients – hence contact isolation – may reduce the number of nosocomial transmissions.

A major challenge to this approach – and generally in rapid infection prevention and control (IPC) – is…

3. The literature survey could be strengthened by reviewing more recent advancements in AI applications in healthcare beyond VRE. This would help situate the study within a broader research landscape.

We have added a broader overview of recent work in digital health (references 25 -32), including references to studies that train convolutional neural networks across various medical domains or use natural language processing to address clinical tasks with AI:

Line 114ff:

Examples include training convolutional neural networks (CNNs) to predict cardiac amyloidosis-suggestive uptake from scintigraphy images^25^, detecting breast artery calcification from mammograms^26^, and estimating carotid artery diameter from ultrasound data^27^. These examples demonstrate how AI can be leveraged for data analysis and pattern recognition in medical applications.

However, beyond analytical capabilities, the interaction between humans and AI is particularly important in medical contexts. Factors such as data privacy, security, and trust perception play a crucial role in the successful adoption of AI-driven solutions. Ensuring that AI tools are interpretable, transparent, and aligned with clinical decision-making processes is essential for their integration into real-world healthcare settings^28,29^.

Additionally, techniques from natural language processing (NLP) have recently attracted interest in the field. Examples include early diagnosis of periodontitis using the language model BERT^30^, or evaluating the performance of large language models (LLMs) on triage and diagnostic accuracy given clinical case vignettes^31^ and clinical information extracting from health records^32^.

We have added two passages for better clarity:

Line 127f:

In the domain of epidemiology, where our work is located, … for their capabilities in …

4. Certain aspects, such as how patient movement data were collected and processed are unclear. More information on data preprocessing steps would enhance reproducibility.

We have added information on how the patient movement data were extracted from the patient data management system records and how we subsequently used them to define unique spatial units as well as their relationships. Details can be found in Sections “Data Collection” and “Representation as a Graph”.

Line 406ff:

Patient movements, which form the foundation of the underlying graph data structure, can be directly extracted from the patient data management system. As the system contains information about patient transfers between wards and rooms, we can use this information to represent spatial interactions within the graph. By continuously modeling this discrete temporal data, we create a detailed representation of patient movement paths over time.

Line 428ff:

Initially, we constructed a static graph model of the hospital, incorporating all spatial units and their connections while excluding patient-related information. This information is derived by first identifying all unique spatial units from the complete list of patient movements extracted from the patient data management system. Relationships between these units can then be identified by analyzing co-occurrences of spatial (sub)levels in the data—for example, room numbers associated with the same ward numbers.

5. The approach to labeling patients as VRE-positive, negative, or unknown is not fully transparent. A clearer explanation of how unknown statuses were treated in model training and evaluation would improve understanding.

We have expanded our description of how we use patient node labels for training the model in the section 'Labeling':

Line 472f:

It should be noted that no distinction is made here between VRE colonization and VRE infection.

Line 475ff:

This means that during training, the weights of the neural network are adjusted solely based on the loss incurred when predicting the VRE carrier status of patients labeled as positive or negative. While the model also predicts a positive/negative label for patients we consider unknown, this information is discarded, as we cannot reliably compare these predictions to a ground truth. The same applies to evaluation.

6. The methodology validation techniques used to assess model performance beyond macro F1 scores, including cross-validation or external validation methods are poor.

We have included additional details on recommended machine learning model evaluation practices in the medical domain and contextualized our evaluation approach with the most relevant studies from related work (references 41 – 42 and 51 - 52):

Line 521ff:

We note that using cross-validation and temporal splits with independent training, evaluation, and test sets is a well-established approach for the robust evaluation of machine learning models in medicine^51,52^. Furthermore, our evaluation extends beyond previous work in the field, which relied on a single fixed train-test split^41^ or a single train-evaluation-test split^42^.

7. There is a lack of visual aids (e.g., performance metrics graphs) that could enhance comprehension of model efficacy. More figures illustrating key findings would be beneficial.

We have added a Boxplot (Figure 4) that visualizes the whole set of results in the Results Section:

Line 208:

Figure 4 provides a graphical comparison of the F1 scores between the static and dynamic models.

8. Providing context on how sensitivity and specificity compare to existing methods would add depth.

To provide a more comprehensive comparison with prior work, we added a paragraph to the ‘Discussion’ section that contextualizes our results in relation to those of related studies.

Line 305ff:

When comparing the sensitivity and specificity scores from these two studies to our results, only Gouareb *et al.*^42^ reported these metrics, whereas Van Niekerk *et al.*^41^ focused on ward-level predictions and reported on the area under the curve (AUC) metric instead. Gouareb et al.’s best-performing setup demonstrated comparable performance (sensitivity = 0.806, specificity = 0.966) when addressing a broad range of multidrug-resistant bacteria. However, different bacteria have variable transmission pathways, patient populations at risk and pathogenicity, making the feasibility of this score insufficient for the evaluation of VRE in particular due to a lack of precision. While our experimental setup is different to this work (we focus on VRE as one type of multi-resistant bacteria and predict infections up to three days in advance) it places our findings in line with prior research and considers the specific insight into the characteristics of this particular pathogen.

9. A more thorough acknowledgment of limitations would strengthen the paper's credibility. Besides, suggestions for how hospitals might implement AI tools based on findings would provide practical value.

In response to the reviewer’s suggestion, we have added a discussion of the limitations in the ‘Limitations’ section, as well as practical implications at the end of the ‘Discussion’ section. Additionally, to provide a more comprehensive comparison with prior work, we included a paragraph that contextualizes our results in relation to those of related studies.

Line 340ff:

Overall, we highlight that this study serves as a proof of concept, which entails in a number of limitations that need to be addressed in future research. Below, we outline these limitations in detail and provide an outlook on how we plan to address them in future work.

Line 336ff:

Additionally, our dataset includes data from only a single hospital, covering only one-third of the patients hospitalized during the one-year observation period. To mitigate this limitation, we employed cross-validation (for the static model) and temporal splits (for the dynamic model) to establish a more robust evaluation setting, demonstrating that our approach yields promising results. However, we emphasize that future research should expand on our work by including a larger and more complete sample of patients over an extended period (e.g., by examining multiple years instead of just one) and incorporating data from additional hospitals to improve the generalizability of our findings.

While our study focused exclusively on VRE, one of the most critical multidrug-resistant bacteria, our approach can be adapted to target other types of bacteria by modifying the target labels. However, its performance in such settings needs to be evaluated to determine whether it is comparable to the results we observed with VRE. For the method to be effectively applied, a certain minimum number of carriers of the microorganism in question must likely be present and known. Otherwise, the system would lack sufficient patterns for learning, which are essential for making reliable predictions. As a result, the method may not be applicable to every potential nosocomial pathogen.

Another limitation of the dataset used in our study is its retrospective nature, which may introduce bias into the modeling process, for example, by including diagnoses that might not be fully available in real-time. This emphasizes the importance of evaluating model performance in predicting VRE carrier status using prospective, real-time data that are automatically limited to information available up to the current day. This would also enable assessing whether performance in such a setting aligns with the results we observed in our study.

Line 315ff:

Future work should focus on deploying our system in a “live” setting within the ICP workflow, which leads us to discuss practical implications and alignment with existing hospital processes. The question of what constitutes an ideal interface between a human and an AI application has attracted a lot of attention recently and the answer to that question clearly depends on each individual use case43. As outlined in the Methodology, a GPU with 6GB of VRAM is sufficient to retrain our proposed model daily (even with increasing graph complexity, e.g., a higher number of patients) to maintain an up-to-date predictor. The most challenging part of setting up the system is integrating existing hospital information systems, which house the relevant data, with the graph representation upon which our algorithm is based. This might require manual effort by technical and medical experts in individual environments. However, once the relevant data are matched, the remaining steps of model training and infection prediction are straightforward. After deployment, another challenge is making the system easily accessible to IPC personnel. The ideal solution may involve developing a user-friendly interface that bridges hygiene experts responsible for screening with the algorithm predicting at-risk patients. However, developing such an interface requires additional effort, such as identifying an easily accessible and user-friendly presentation of the information or ensuring that there is no misinterpretation of the algorithm’s screening recommendations. These can then serve as guidance for but not replace IPC team.

10. Simplifying language where possible could enhance understanding without sacrificing scientific rigor.

We hope that the changes already made in response to the comments above have led to some improvements. Additionally, we have made the following revisions in the Materials and Methods section:

Line 428ff:

~~Initially, we created a static graph representation of the "uninhabited" hospital involving all hospital-related node sets and connecting edge types, excluding patient relations.~~

Initially, we constructed a static graph model of the hospital, incorporating all spatial units and their connections while excluding patient-related information.

Line 459f:

~~We modeled the spatial relations of patient movements on a daily basis, generating a distinct graph G for each day T that reflects the spatial relations present at that time.~~

To account for temporal dynamics, we created a separate graph for each day, capturing the spatial relations and patient movements occurring on that specific day.

—

Reviewer #2: The manuscript presents a compelling exploration of how AI, and in specific GNNs, can be employed to address a critical challenge in healthcare, mainly contributing to the early identification of vancomycin-resistant enterococci (VRE) carriers. This research is situated at the intersection of computational modeling and infection prevention, an area of growing importance given the global rise in antimicrobial resistance. By leveraging hospital data to model patient interactions and spatial dynamics, the study offers a data-driven approach to predicting VRE status, with the potential to transform current infection control practices.

Methodological Evaluation: This study introduces a sophisticated application of GNNs to predict the presence of VRE carriers in a hospital setting. Methodologically, the research distinguishes itself by employing two models: a static model that aggregates patient movement data without temporal dynamics, and a dynamic model that integrates temporal graph learning. The dynamic model, which contextualizes patient interactions over time, is particularly noteworthy and outperforms the static model in all evaluated metrics as it showed in the submission. The reliance of this study on time-dependent graph sequences to model a "living hospital" environment is certainly an innovative approach per se, and it addresses a critical gap in infection prevention and control research. The inclusion of heterogeneous data points, such as ward and room locations, patient movements, and ICD/OPS codes, reflects an advanced integration of multidimensional data for predictive modeling. The application of advanced temporal learning techniques further enhances the methodology, offering predictive capabilities that could significantly benefit infection control protocols in real-world clinical settings. However, there are limitations in the methodology that warrant attention, and mainly raising concerns on the generalizability of its outcomes, as the training data were collected and used from a single hospital, and the secondly, the relatively small sample of confirmed VRE cases (247 patients) may constrain the robustness of the findings. While the use of cross-validation partially mitigates these limitations, future iterations of the study should include multi-center datasets and explore external validation to strengthen its applicability.

Thank you for your feedback! We have added the following passage to the Limitations section:

Line 346ff:

Additionally, our dataset includes data from only a single hospital, covering only one-third of the patients hospitalized during the one-year observation period. To mitigate this limitation, we employed cross-validation (for the static model) and temporal splits (for the dynamic model) to establish a more robust evaluation setting, demonstrating that our approach yields promising results. However, we emphasize that future research should expand on our work by including a larger and more complete sample of patients over an extended period (e.g., by examining multiple years instead of just one) and incorporating data from additional hospitals to improve the generalizability of our findings.

Line 367ff:

As a final and significant limitation, we would like to highlight the uncertainty of the labeling due to the limited sensitivity of VRE screening tests. The detection rate of rectal swabs for identifying VRE carriers has been reported to be as low as 58%, depending on the bacterial density in stool samples^44^. Consequently, some VRE-positive patients in our dataset may have been misclassified as negative, potentially affecting the quality of the labels used for model training. Additionally, early-stage colonization may not be detected by a single test, necessitating serial sampling for accurate identification45. This diagnostic uncertainty in screening strategies could impact the model’s performance, and future studies should consider its potential influence on predictive accuracy.

Scientific Contribution and Value: The manuscript contributes meaningfully to the field of healthcare-associated infection prevention by advancing predictive analytics for VRE carrier identification. The incorporation of GNNs into hospital data modeling helped the study in addressing the inherent complexity of VRE transmission pathways, which involve both direct patient interactions and environmental contamination. The predictive framework, which anticipates VRE status up to three days in advance, is particularly impactful, as it provides infection control teams with a temporal advantage to implement preventive measures. On another note, this research builds upon and extends prior studies by leveraging heterogeneous graph structures that capture detailed environmental variables, such as beds, wards & rooms, and temporal patient trajectories. Unlike these earlier studies, which were limited to retrospective classification or homogenous graph models, the current approach offers a dynamic and forward-looking perspective, positioning it as a potential cornerstone for automated infection prevention systems. Despite its evident value, the study's real-world utility would be significantly enhanced by a more detailed discussion on its scalability and integration into existing hospital workflows. Additionally, the lack of a thorough cost-benefit analysis leaves open questions about its feasibility in resource-constrained settings.

We have thought about this and written it down in the discussion:

Line 315ff:

Future work should focus on deploying our system in a “live” setting within the ICP workflow, which leads us to discuss practical implications and alignment with existing hospital processes. The question of what constitutes an ideal interface between a human and an AI application has attracted a lot of attention recently and the answer to that question clearly depends on each individual use case^43^. As outlined in the Methodology, a GPU with 6GB of VRAM is sufficient to retrain our proposed model daily (even with increasing graph complexity, e.g., a higher number of patients) to maintain an up-to-date predictor. The most challenging part of setting up the system is integrating existing hospital information systems, which house the relevant data, with the graph representation upon which our algorithm is based. This might require manual effort by technical and medical experts in individual environments. However, once the relevant data are matched, the remaining steps of model training and infection prediction are straightforward. After deployment, another challenge is making the system easily accessible to IPC personnel. The ideal solution may involve developing a user-friendly interface that bridges hygiene experts responsible for screening with the algorithm predicting at-risk patients. However, developing such an interface requires additional effort, such as identifying an easily accessible and user-friendly presentation of the information or ensuring that there is no misinterpretation of the algorithm’s screening recommendations. These can then serve as guidance for but not replace IPC team.

Discussion and Conclusions: The authors articulate the strengths of their model with clarity, emphasizing its capacity to predict VRE status dynamically and its superiority over static models. The discussion effectively contextualizes the findings within the broader literature on AI applications in infection control, demonstrating both the novelty and relevance of the proposed methodology. However, the manuscript could benefit from a more critical evaluation of its limitations, particularly regarding the potential biases introduced by the single-center dataset and the reliance on retrospective data.

We have now addressed this point in the limitations:

Line 346ff:

Additionally, our dataset includes data from only a single hospital, covering only one-third of the patients hospitalized during the one-year observation period. To mitigate this limitation, we employed cross-validation (for the static model) and temporal splits (for the dynamic model) to establish a more robust evaluation setting, demonstrating that our approach yields promising results. However, we emphasize that future research should expand on our work by including a larger and more complete sample of patients over an extended period (e.g., by examining multiple years instead of just one) and incorporating data from additional hospitals to improve the generalizability of our findings.

Line 361ff:

Another limitation of the dataset used in our study is its retrospective nature, which may introduce bias into the modeling process, for example, by including diagnoses that might not be fully available in real-time. This emphasizes the importance of evaluating model performance in predicting VRE carrier status using prospective, real-time data that are automatically limited to information available up to the current day. This would also enable assessing whether performance in such a setting aligns with the results we observed in our study.

The authors do acknowledge these limitations but stop short of discussing their implications for broader implementation. A more detailed exploration of the practical barriers to real-time deployment, such as computational demands and integration with electronic health record (EHR) systems, would strengthen the manuscript’s impact.

We have added this point in the section mentioned above (line 315ff)

On a different note, the conclusion, while succinct, highlights the study's promise as a tool for early detection of VRE carriers, underscoring its potential to revolutionize current screening practices. By enabling the proactive interventions, this approach could reduce nosocomial transmission rates and improve patient safety. However, the authors should expand their discussion of how this methodology could be adapted to other multidrug-resistant organisms (MDROs) to underline its broader applicability.

We have added this point to the limitation:

Line 354ff:

While our study focused exclusively on VRE, one of the most critical multidrug-resistant bacteria, our approach can be adapted to target other types of bacteria by modifying the target labels. However, its performance in such settings needs to be evaluated to determine whether it is comparable to the results we observed with VRE. For the method to be effectively applied, a certain minimum number of carriers of the microorganism in question must likely be present and known. Otherwise, the system would lack sufficient patterns for learning, which are essential for making reliable predictions. As a result, the method may not be applicable to every potential nosocomial pathogen.

In conclusion, the study introduces several groundbreaking innovations: a) integrating of temporal dynamics into graph modeling which represents a significant methodological advancement, enabling the prediction of patient VRE status with unprecedented accuracy; b) the inclusion of diverse data types, be it spatial, temporal, and clinical, captures the complexity of hospital environments and offers a granular view of VRE transmission pathways; c) the ability to forecast VRE status up to three days in advance is a transformative step forward, offering actionable insights for infection control teams. These features collectively position the study at the forefront of AI applications in healthcare-associated infection control.

Recommendation: Based on the strengths and innovative approaches applied and integrated by this study, and despite the highlighted limitations of the manuscript, I recommend accepting this manuscript for publication, after highlighting the limitations of this study.

—

Reviewer #3: Great paper, in well-described depth as well. It would be helpful to hear more about the "microbiological data" comment which prompted a question in my mind about whether culture data (blood, urine, etc) was included. That would obviously be critical to prevent data leakage in addition to the ICD code excluded, but there is good description elsewhere in the methods section of the other variables included to my satisfaction.

Thank you for your feedback! We expanded on the usage of microbiological data in the Section “Data Collection”:

Line 413ff:

We do not explicitly include microbiological data from the laboratory information system as patient characteristics. However, such information can be to some degree encoded in the ICD codes that are part of the patient features. We will use microbiological data (VRE test results) to assign labels to patients.

—

Reviewer #4: Thanks for the opportunity to read this interesting paper.
The authors explored the benefits of artificial intelligence tools to identify and predict patients at risk of VRE carriage. The purpose is of real interest for IPC practitioners and epidemiologists and deals with the tools and data available due to IT.

The paper is easy to read and well-written.

I think the term “outbreak detection” seems a too much because the aim of the study is to detect VRE carriers, not outbreak.

Thank you for your comment! We understand the concern regarding the term "outbreak detection." As some of the authors work in hospital infection control, we recognize that identifying VRE carriers is a crucial foundation for detecting and preventing outbreaks. We have revised the conclusion to clarify this connection and hope that this adjustment addresses the reviewer's concern.

Line 382ff:

~~Ultimately, this strategy would provide a temporal advantage for IPC teams by enabling the early identification of potential VRE carriers. This could facilitate the implementation of tailored IPC strategies and potentially reduce outbreak scenarios.~~

Ultimately, this strategy would provide a temporal advantage for IPC teams by enabling the early identification of potential VRE carriers. Since unrecognized carriers can contribute to silent transmission, their timely detection is essential for preventing larger transmission chains. By systematically identifying at-risk patients, our approach lays the groundwork for effective IPC strategies and supports outbreak prevention—reinforcing the idea that outbreak prediction starts with carrier identification.

I should be valuable to evaluate the performance of the models on a different year of the hospital or in another hospital. This would reinforce the validity of the models. We're at the feasibility study stage at the moment.

Thank you for your feedback. We expanded on the limitations identified in our research and provide a more comprehensive discussion of these factors in the revised “Limitations” section.

Line 346f:

Additionally, our dataset includes data from only a single hospital, covering only one-third of the patients hospitalized during the one-year observation period.

Line 361ff:

Another limitation of the dataset used in our study is its retrospective nature, which may introduce bias into the modeling process, for example, by including diagnoses that might not be fully available in real-time. This emphasizes the importance of evaluating model performance in predicting VRE carrier status using prospective, real-time data that are automatically limited to information available up to the current day. This would also enable assessing whether performance in such a setting aligns with the results we observed in our study.

The key predictors found in the study are diagnosis and procedures codes. It's consistent with literature, but it's not the more interesting data to make a real-time predictive tool. These data are often available a few days or weeks after patient discharge.

Procedures that demonstrate good performance are available in real-time (the same day, with only minor changes in the billing process) at our hospital. However, we acknowledge that this may not be representative of all hospitals. Regarding the ICD codes, we expanded on the potential bias introduced by the retrospective nature of our study in the Limitations section (line 361ff).

In table 1, I am not sure to understand some terms and theirs meaning: Braden? Barthel? Bedscore? Clinical chemistry from blood? ... I think this should explain in methods.

We have added an explanation of this in Table 1 and the Methodology Section.

Line 442f:

Braden scale - pressure ulcer risk

Line 443:

Barthel index - disability assessment score

In the discussion, I think the contribution of the novel model in real life for IPC should be discussed. As well as the work to do before implementing the model.

We have extended our discussion as follows:

Line 315ff:

Future work should focus on deploying our system in a “live” setting within the ICP workflow, which leads us to discuss practical implications and alignment with existing hospital processes. The question of what constitutes an ideal interface between a human and an AI application has attracted a lot of attention recently and the answer to that question clearly depends on each individual use case^43^. As outlined in the Methodology, a GPU with 6GB of VRAM is sufficient to retrain our proposed model daily (even with increasing graph complexity, e.g., a higher number of patients) to maintain an up-to-date predictor. The most challenging part of setting up the system is integrating existing hospital information systems, which house the relevant data, with the graph representation upon which our algorithm is based. This might require manual effort by technical and medical experts in individual environments. However, once the relevant data are matched, the remaining steps of model training and infection prediction are straightforward. After deployment, another challenge is making the system easily accessible to IPC personnel. The ideal solution may involve developing a user-friendly interface that bridges hygiene experts responsible for screening with the algorithm predicting at-risk patients. However, developing such an interface requires additional effort, such as identifying an easily accessible and user-friendly presentation of the information or ensuring that there is no misinterpretation of the algorithm’s screening recommendations. These can then serve as guidance for but not replace IPC team.
